# Rehabilitation Strategies for Pediatric Single-Sided Deafness, Including Children with Asymmetric Hearing Loss: A Systematic Review

**DOI:** 10.3390/audiolres16050129

**Published:** 2026-09-01

**Authors:** Daniele De Seta, Elisabetta Oliva, Stefano Manca di Villahermosa, Pietro De Luca, Patrizia Mancini, Hilal Dincer D’Alessandro, Fernanda Asprella-Libonati, Rosaria Turchetta, Francesca Yoshie Russo

**Affiliations:** 1 Unit of Otolaryngology and Audiology, Department of Sense Organs, Sapienza University of Rome, 00185 Rome, Italy; daniele.deseta@uniroma1.it (D.D.S.); p.mancini@uniroma1.it (P.M.); hilal.dincer@uniroma1.it (H.D.D.); rosaria.turchetta@uniroma1.it (R.T.); francescayoshie.russo@uniroma1.it (F.Y.R.); 2 Unit of Otolaryngology, Department of Surgery, CTO Hospital, ASL Sulcis, 09016 Iglesias, Italy; stedivilla94@gmail.com; 3 Department of Otolaryngology, San Giovanni-Addolorata Hospital, 00184 Rome, Italy; dr.dlp@hotmail.it; 4 Independent Researcher, 75100 Matera, Italy; fernandasprella@gmail.com

**Keywords:** binaural hearing, bilateral hearing, cochlear implant, BAHA, bone conduction, hearing aid, CROS, SSD, asymmetric hearing loss, unilateral hearing loss, children

## Abstract

**Objectives:** This study aimed to analyze in a comprehensive systematic review the hearing rehabilitation strategies for single-sided deafness (SSD) and asymmetric hearing loss (AHL) in children. Current rehabilitative options include contralateral routing of signal (CROS) devices, bone conduction devices, and cochlear implantation (CI). **Methods:** A systematic review of PubMed/MEDLINE was performed to identify studies on pediatric SSD and AHL rehabilitation published up to 7 July 2025. Reviews, meta-analyses, case reports, and animal studies were excluded. Eligible studies included pediatric populations treated with rehabilitative devices, with outcomes focused on speech perception, auditory performance, binaural hearing, quality of life, and questionnaire-based measures. Study selection followed PRISMA principles. **Results:** A total of 1527 records were identified by three authors through PubMed/MEDLINE (2003–July 2025) after duplicate removal. Title screening retained 581 studies, and abstract screening retained 267 studies. Fifty-eight full-text papers were assessed for eligibility; 10 were excluded due to overlapping patient cohorts, leaving 48 papers for the qualitative synthesis. Overall, 1622 children were analyzed; 43 studies focused on SSD and five on AHL. **Conclusions:** Available evidence supports rehabilitation in pediatric SSD and AHL. CROS systems offer a non-surgical and reversible option with benefits in selected listening situations but limited effects on localization. Bone conduction devices may improve hearing in noise and some functional outcomes, especially in conductive indications. CI appears to provide the greatest opportunity to restore bilateral auditory input, particularly when performed early in congenital SSD.

## 1. Introduction

Binaural hearing is the normal physiological condition in which a sound stimulus is perceived by the two ears and subsequently processed and integrated at peripheral and central levels. Auditory stimulus analysis systems and coordination between the two sides provide several advantages over unilateral hearing. The head shadow effect mechanism (peripheral), binaural summation, and signal segregation from noise or squelch effect (central) mainly allow accurate spatial localization of the sound source (which reach a minimum of 1 degree in the horizontal plane for frequencies around 500 to 750 Hz), better understanding of speech in noise and reverberation, and, in general, greater ease of listening and development of spoken language [1,2].

These phenomena rely on binaural cues such as the interaural time difference (ITD) and interaural level difference (ILD). Sources displaced to the side in the horizontal plane reach the near ear earlier and at higher intensity than the far ear. For amplitude-modulated signals such as speech, ITD cues are also available from differences in the timing of the envelopes of the stimuli. In ILD, the head acts as an acoustic shadow reducing the intensity of the sound reaching the ear farthest from the source. The use of ITD cues is mainly restricted to low frequencies because of temporal-resolution limits, whereas ILDs are larger at high frequencies [3].

A large number of studies have demonstrated the importance of binaural cues for sound-source localization and for improving the perception of target sounds in the presence of competing sounds [4]. By eliminating binaural cues or modifying the relationship between interaural acoustic differences and directions in space, unilateral or asymmetric hearing loss can have a disruptive impact on spatial hearing. The lack of binaural cues and reduced audibility negatively affect communication and quality of life [5]. Furthermore, monaural deprivation in infancy can induce maladaptive changes in the brain that may persist even if hearing in the affected ear is restored, resulting in longer-term deficits in spatial hearing [6]. At the same time, there is growing evidence that plasticity of central auditory processing can partially compensate for hearing loss in one ear, leading to some recovery in sound-localization ability [7,8].

The estimated incidence of sensorineural hearing loss greater than 40 dB at birth is 1.86 per 1000 newborns in developed countries, and 30% to 40% of these cases are unilateral [9,10]. In school age, the incidence of unilateral hearing loss, whether sensorineural or transmissive and of any degree, is estimated to be between 3% and 6% of children [11]. A growing number of studies show that children with unilateral hearing loss not only have difficulty localizing sound and understanding speech in noise but may also have difficulty mastering complex spoken language skills [12]. Even mild hearing loss places a child at risk for lasting language delays, although speech disorders are not an inevitable consequence of hearing loss [13].

Some studies report that 13% of children with unilateral severe-to-profound hearing loss or single-sided deafness need individual school support, and up to 35% may repeat a school year [14,15]. On intelligence quotient (QI) tests, children with SSD may score lower than peers with normal hearing or with mild or moderate hearing loss. Lower school results in children with SSD may be attributable to reduced verbal discrimination in noise, poorer localization ability, and decreased concentration due to increased cognitive effort required to compensate for the hearing deficit. This may be amplified by the large amount of time children spend in acoustic unfavorable environments, with high levels of background noise and reverberation [16].

Indeed, the ability to understand degraded language is crucial in developmental processes that last until adolescence [17]. Moreover, several studies have shown that the need to spend energy to focus attention on the verbal message leads to a lower availability of cognitive resources and therefore to a decrease in performance in contexts of multi-activity and learning [18].

In the past two decades, universal newborn hearing screening (UNHS) has been widely adopted throughout North America, Europe, and most other developed regions, primarily as a result of technological advances in screening and intervention modalities. UNHS has been part of the essential levels of care in Italy since 2007. This screening program has allowed earlier diagnosis of congenital hearing loss and timelier management of hearing rehabilitation [19]. Although brain plasticity is present throughout life, its maximum expression and effectiveness occur during the first years [20].

Following the diagnosis of severe-to-profound bilateral hearing loss, rehabilitation with cochlear implantation, which represents the gold-standard treatment for this type of deficit, is offered toward the first year of life to allow exposure to sound stimuli for auditory pathway development and subsequent speech and language development. Most children with bilateral congenital deafness and two cochlear implants obtain superior performance in verbal comprehension and language development compared with subjects with the same deficit implanted on one side only; furthermore, younger age at implantation is associated with better auditory performance [1,21]. Restoration of bilateral stimulation at a young age may therefore also be beneficial for children with unilateral severe-to-profound hearing loss.

Mild and moderate sensorineural hearing loss in children is often managed with hearing aids, whereas transmissive hearing loss can be treated with bone-transmission hearing implants, either passive or active [22,23]. Before the diffusion of UNHS, detection of unilateral hearing impairment in children was often accidental, with ascertainment typically occurring around five or six years of age [24,25,26]. UNHS has clearly lowered the detection age of childhood hearing impairment, with age at diagnosis in screened newborns becoming similar between unilateral and bilateral cases [27].

Over the past decade, an increasing number of adults and, more recently, children with severe-to-profound unilateral sensorineural hearing loss have been treated with cochlear implants. In 2024, an update of national Italian guidelines reportedly supported CI for selected patients with single-sided deafness, recommending multidisciplinary audiological evaluation and follow-up, in line with the growing body of clinical studies showing benefit.

The difference between adults and children is that in adults the CI is generally applied to SSD acquired in post-lingual age, and therefore acts on an already developed auditory pathway that must be restored; in children, the implant has the role of stimulating the ipsilateral auditory pathway that otherwise would not have developed adequately. Therefore, the importance of early diagnosis and rehabilitation lies in allowing central plasticity to support the development of the auditory pathway and maximize correct binaural or bilateral hearing development. The results reported by pediatric studies are often variable, presumably because of small and heterogeneous samples in terms of age of onset and age at implantation. Even less evidence exists on the effectiveness of rehabilitation in moderate unilateral deafness.

Furthermore, much of the available research focuses on spatial localization abilities and audiological advantages of binaural hearing rather than on neurocognitive factors such as language production and executive functions. To make evidence-based decisions regarding whether and when to rehabilitate children with SSD, it is important to consider not only the audiological consequences of unilateral hearing loss but also its functional and neural effects, as well as the time window for optimizing the child’s performance. 

The present review aims to describe the state of the art of management of congenital or peri-lingual unilateral hearing loss in children, considering both SSD and AHL.

## 2. Materials and Methods

### 2.1. Study Design

A comprehensive review was performed by researching all articles in the PubMed/MEDLINE database from database inception in 2003 until 7 July 2025.

### 2.2. Eligibility Criteria

The search strategy excluded reviews, meta-analyses, case reports, and animal studies. The following study types were included: randomized controlled trials (blinded and/or placebo-controlled), non-randomized prospective studies, and retrospective chart reviews. Three authors (SMdV, DDS and EO) screened and selected papers to be included in the study. Only English-language reports were included. No exclusion based on outcomes was applied.

### 2.3. Outcomes of Interest

The principal outcomes considered in the literature were speech perception score, auditory outcomes, quality of life, Children’s Home Inventory for Listening Difficulties (CHILD), Abbreviated Profile of Hearing Aid Benefit (APHAB), binaural summation, squelch, head shadow effect, tinnitus handicap index (THI), Speech, Spatial and Qualities of Hearing Scale (SSQ), and self-administered questionnaires.

### 2.4. Study Selection

**Figure 1:**
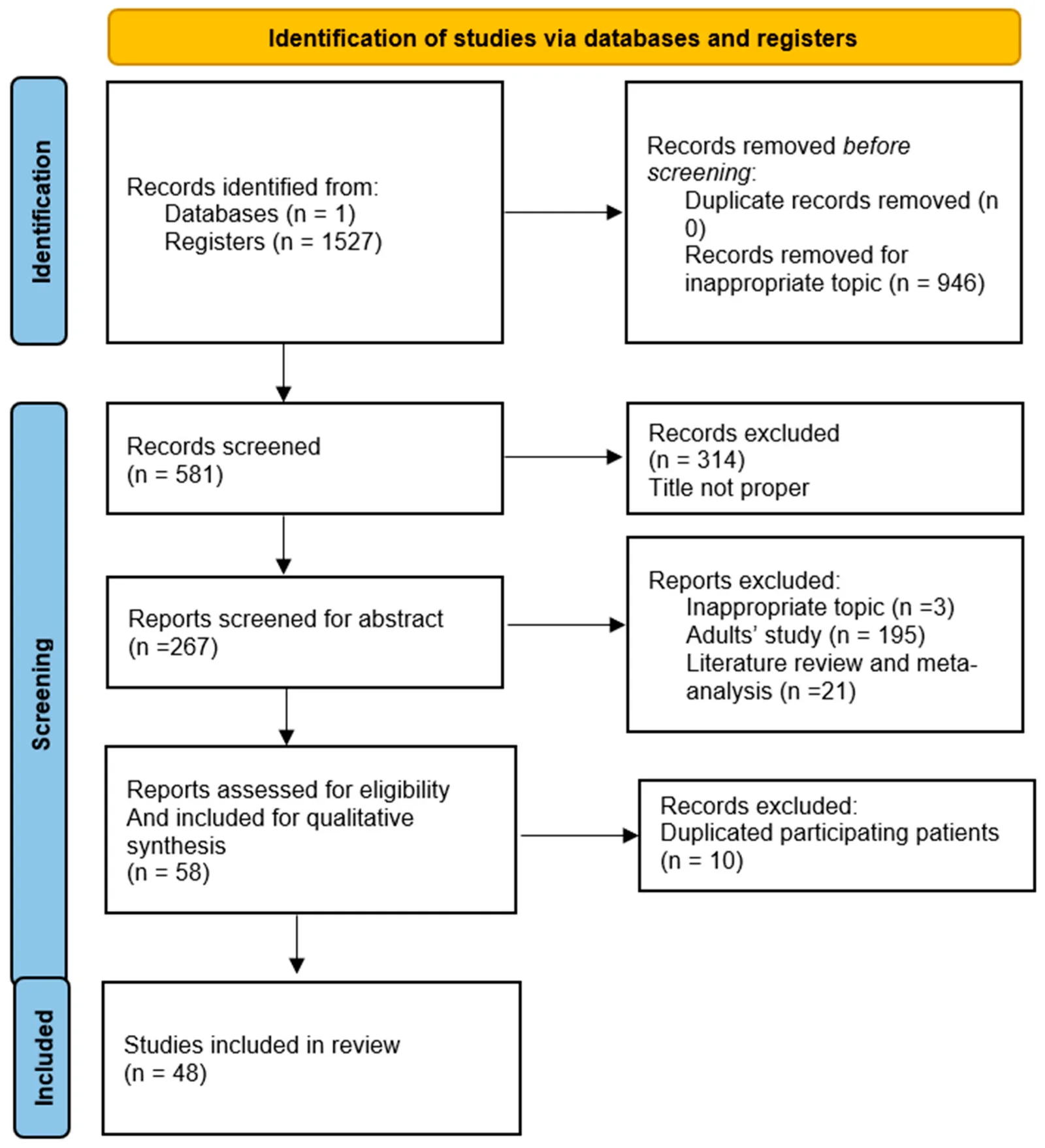
**PRISMA** (Preferred Reporting Items for Systematic Reviews and Meta-Analysis) flow diagram for study selection.

**Figure 2:**
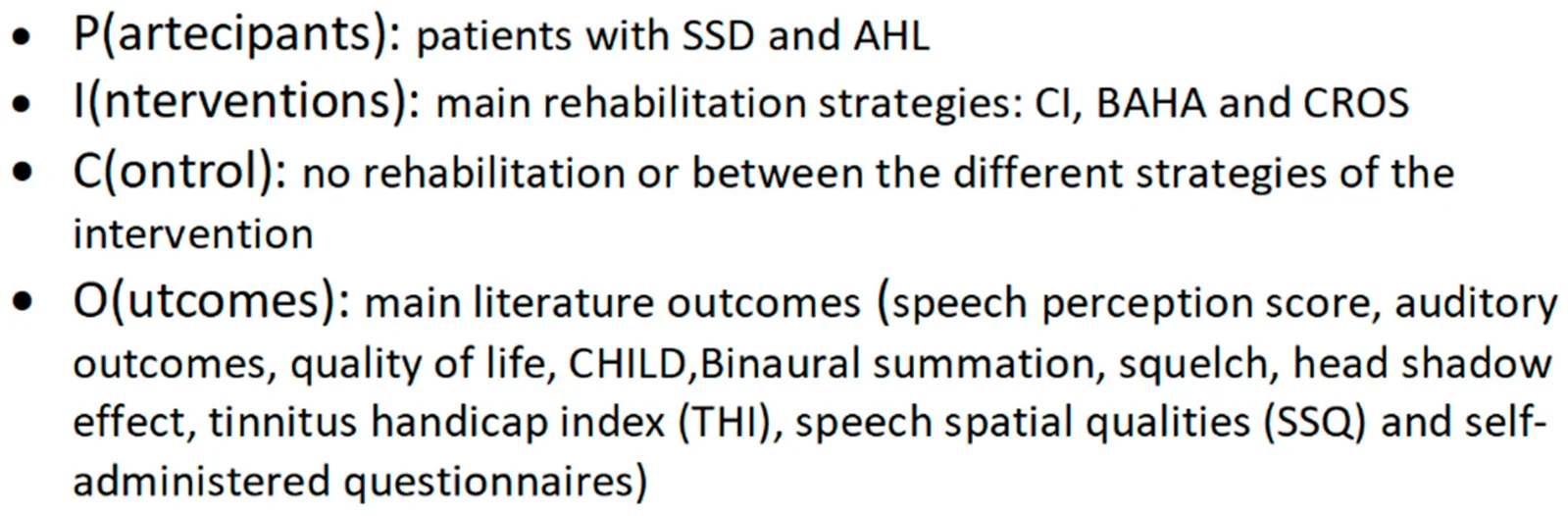
PICO framework. SSD: single-sided deafness, AHL: asymmetric hearing loss, CI: cochlear implant, BAHA: bone-anchored hearing aid, CROS: contralateral routing of signal, Abbreviated Profile of Hearing Aid Benefit, THI: tinnitus handicap index, speech spatial qualities (SSQ).

The search was conducted according to PRISMA (Preferred Reporting Items for Systematic Reviews and Meta-Analyses) principles as reported in Figure 1 [28]. The main research objects were listed according to the PICO framework, as reported in Figure 2. The PubMed/MEDLINE database was systematically searched using the following MeSH terms and free-text string:

((“Hearing Loss, Unilateral” [Mesh]) OR (“single-sided deafness”) OR (“single sided deafness”) OR (“asymmetric hearing loss”) OR (“asymmetric sensorineural hearing loss”)) AND ((((“Contralateral routing of signal”) OR (“Contralateral-routing of signal”)) OR ((“Osseointegration” [Mesh]) OR (“Bone Conduction” [Mesh]) OR (“Prosthesis Implantation” [Mesh]) OR (“Hearing Aids” [Mesh]) OR (“BAHA”) OR (“Bone anchored hearing aid*”) OR (“Bone-anchored hearing aids”) OR (“Bone anchored devic*”) OR (“Bone anchored aid*”) OR (“PONTO”) OR (“Bone conduction implant*”))) OR ((“Cochlear Implants” [Mesh]) OR (“Cochlear Implantation” [Mesh]) OR (“cochlear impl*”))).

Three authors screened and selected papers to be included in the study. A manual review subsequently selected studies including pediatric-only subjects and mixed populations, excluding those with adult-only subjects. Studies with overlapping patients were excluded by maintaining the most recent follow-up; similarly, studies involving the same single patients in different reports were handled to avoid duplication. Overlap was identified by comparing the series of patients enrolled in studies from the same center and by author matching, considering the most recent publication and evaluating patient age to ensure that duplicate cohorts were not included. No publication-date restriction was imposed.

Study selection and quality assessment were conducted independently by the reviewers and were based solely on predefined eligibility criteria without consideration of publication status or potential impact on the review findings.

Following study selection, the methodological quality of the included non-randomized studies was assessed using the Newcastle–Ottawa Scale (NOS). A standardized quality assessment table (Table S1) was prepared to evaluate the risk of bias across the included studies.

## 3. Results

### 3.1. Search Results

After duplicate removal, the search found a pool of 1527 works through PubMed/MEDLINE, published between 2003 and July 2025. Title-based selection identified 581 studies, and subsequent abstract screening selected 267 papers. Fifty-eight full-text papers were assessed for eligibility, ten papers were excluded because of duplicated patients participating in multiple studies, and finally 48 papers were included in the qualitative synthesis. A total of 1622 children were included in the analysis, 43 papers focused on rehabilitation of SSD, and five papers concerned AHL rehabilitation.

Ten studies had a prospective design, none was a randomized controlled trial, and the remaining studies were retrospective chart reviews.

### 3.2. Studies on SSD

Concerning SSD papers, 32 studies reported CI rehabilitation outcomes, 10 reported BAHA outcomes, two reported CROS outcomes, one reported active middle ear implant outcomes, and two reported mixed modalities including CI, BAHA, and CROS. A total of 903 children underwent cochlear implantation, 479 were rehabilitated with BAHA systems, and 54 with CROS. Early rehabilitation with CI before the age of 4 years was performed in 395 patients. Among subjects rehabilitated with BAHA or AMEI, 53 children had sensorineural hearing loss and 42 were implanted because of conductive hearing loss associated with congenital malformations.

### 3.3. Studies on AHL

The 135 children included in the AHL studies were almost all bimodal users (CI plus hearing aid). Twenty-two patients had congenital hearing loss. The etiology of deafness was not specified for all children *(n* = 49) in the study by Perkins et al., while most of them appeared to have congenital onset [29]. Information about early cochlear implantation was reported by Tzifa et al. in three children and cannot be extrapolated from Schaefer et al., where the mean age at CI was 4.9 years (range 1.1–14.2) in a population of 57 children [30,31].

### 3.4. Main Findings Across Modalities

Among all the selected papers, only one study analyzed different rehabilitation modalities without comparing outcomes between groups [32]. Some other papers reported preliminary hearing-aid trials before cochlear implantation and soft-band BAHA or CROS trials. All the other reports evaluated outcomes of a single rehabilitation modality, mainly retrospectively [33].

Spatial-recognition tests were often the core tests in these studies, providing useful information on binaural or pseudo-binaural function in rehabilitated children. However, these test settings were structured in a non-standardized fashion; distance between speakers and listener, intensity of the signal, and other details were frequently not reported in detail. Audiological tests used were not homogeneous. The most often reported were pure-tone audiometry, signal-to-noise ratio tasks, Oldenburg Sentence Tests, consonant–nucleus–consonant tests, Arizona Biomedical sentence testing, and HINT. Perceived quality-of-hearing and quality-of-life tests usually utilized were APHAB and SSQ. Causes of deafness were not consistently reported. Demographic, etiological, clinical, and audiological features of the entire population are reported in Table 1.

Audiological outcomes were reported to improve after treatment regardless of rehabilitation type in most cases. Improvement in binaural functions, such as sound–space recognition, was also described. These features improved more in CI rehabilitated children than in BAHA or CROSS cohort. As a general measure of CI outcome and acceptance, most children reportedly wore their speech processor regularly and throughout the day. Smaller children showed awareness of the sound processor and requested new batteries or repositioning of the coil [34].

## 4. Discussion

The present review demonstrates that SSD and AHL in pediatric patients have become topics of growing interest within audio-otology. The earliest selected study dates back more than a decade [35]. Since the introduction of cochlear implantation as a potential treatment for tinnitus in adults with SSD, increasing attention has been devoted to determining whether this rehabilitative approach is effective in children as well [36,37]. As summarized in this review, audiological data from more than 1600 children with SSD and AHL treated with various modalities have now been published.

In the past, SSD and unilateral hearing loss were considered minor conditions under the assumption that a single hearing ear was sufficient to perform all auditory tasks. However, numerous studies in recent years have demonstrated that school-aged children with unilateral hearing loss exhibit substantial reductions in speech perception in noise and poorer hearing-related quality of life [38]. Before the introduction of CI, SSD and AHL were commonly managed using bone conduction devices, which proved highly effective in cases of conductive hearing loss, such as congenital aural atresia, where cochlear function was preserved.

In one cohort, 100 children were rehabilitated with bone-anchored devices, more than one-third of whom presented with congenital auricular atresia [39]. Favorable audiological outcomes were observed, consistent with Nelissen et al., who reported long-term improvements in both SSQ and PTA scores in patients with unilateral conductive hearing loss treated with BAHA, including 20 children with congenital auricular atresia [23]. Despite these encouraging audiological results, poor compliance was noted, suggesting that careful selection of candidates is mandatory.

The introduction of CI for SSD and AHL has represented a breakthrough. The possibility of restoring binaural hearing and achieving favorable outcomes in implanted children has encouraged many centers to adopt this rehabilitative approach, particularly for sensorineural etiologies. Despite the enthusiasm of clinicians and families, strong evidence demonstrated CI superiority over alternative interventions remains limited, and early implantation for congenital SSD still falls outside candidacy criteria in most countries. The considerable heterogeneity of testing protocols and the inherent difficulty in assessing binaural function are key barriers to establishing definitive conclusions. The CROSSSD study by Katiri et al. represented one of the first attempts to standardize outcome domains for SSD, but further child-centered guidelines are necessary to enable robust statistical comparisons and standardized methodologies in future studies [40].

Studies in both adults and children have shown that the ability to segregate target speech from competing speech or noise in complex auditory environments depends on a combination of monaural and binaural computations using spatial and head-shadow cues [4,41,42,43,44,45]. Spatial cues, in particular, play a pivotal role in source segregation, with speech intelligibility improving markedly when target and competing sounds are spatially separated, a phenomenon known as spatial release from masking [41,46,47,48,49,50]. This benefit becomes even more pronounced in acoustic complex environments, where informational masking makes spatial cues crucial for effective auditory-scene analysis [51,52,53,54].

### 4.1. Congenital vs. Acquired Unilateral Hearing Loss

#### Auditory Pathway Development in SSD

Auditory pathway development depends on continuous sensory stimulation; therefore, in congenital or perinatal deafness, sensory deprivation profoundly alters functional connectivity within the auditory system. Deprivation during sensitive developmental periods disrupts cortical maturation, resulting in degraded auditory processing and impaired behavioral performance. Accordingly, clinical management of pediatric hearing loss must consider the status of central auditory maturation. When a congenitally deaf child receives appropriate auditory stimulation within the critical window, normal cortical organization and age-appropriate spoken-language development are achievable [20,55,56,57]. Conversely, delayed auditory input beyond this period leads to atypical cortical reorganization, in which auditory regions are co-opted by visual or somatosensory modalities, diminishing later auditory function [20,55].

Multiple studies have reported poor auditory outcomes in late-implanted children, likely reflecting cross-modal plasticity, in which other sensory modalities recruit auditory cortical areas. In congenital SSD, early auditory deprivation leads to maladaptive cortical reorganization known as aural preference syndrome, in which the normal-hearing ear dominates cortical representation and the deprived pathways remain weakly encoded.

CI in young children with SSD promotes auditory pathway development by restoring bilateral input and enabling maturation of binaural hearing. Polonenko et al. and Perkins et al. demonstrated rapid reestablishment of symmetric cortical representation in young children implanted for congenital unilateral deafness, suggesting that early CI use can normalize auditory cortical function [58,59].

Arndt et al. and Beck et al. proposed that implantation before the age of four yields optimal outcomes in congenital SSD [60,61]. Subsequent work identified 3 years and 2 months as a more precise upper limit. Early implantation appears to reverse the asymmetric cortical activation characteristic of aural preference [55]. Conversely, higher rates of non-use and poorer outcomes have been observed by Tavora-Vieira et al. among late-implanted patients [62]. In the present literature, both congenital and acquired SSD patients demonstrate improvements in speech perception following CI, but the best outcomes occur in congenitally deaf children implanted before age four.

It remains unclear whether the contralateral normal-hearing ear provides partial cortical protection in SSD by maintaining bilateral auditory pathway activation. Nevertheless, electrophysiological data indicate that sustained asymmetric hearing can lead to aural preference syndrome, in which cortical reorganization favors the better-hearing ear and suppresses binaural processing. Other reports, however, show that even late-implanted congenital SSD children may consistently use their CIs. Three congenital late-implanted children showed limited or no speech perception in quiet with the CI side only, although sound localization and speech perception in noise improved significantly. Subjectively, both parents and children reported better performance in everyday life and school.

### 4.2. True Binaural vs. Pseudo-Binaural Hearing Rehabilitation

CROS hearing aids are effective in enhancing the signal-to-noise ratio when the sound source is located on the side of hearing impairment; however, they may reduce signal-to-noise ratio in other scenarios [63]. Significant improvements in noise performance as measured by HINT, as well as enhanced satisfaction measured with the CHILD questionnaire, have been observed in children with profound unilateral sensorineural hearing loss [35].

Studies evaluating CROS systems in children with simulated unilateral hearing loss have demonstrated that these systems can improve sentence recognition and story comprehension in noisy classroom environments compared with unaided conditions and remote microphone configurations [64]. In a study involving 20 children with SSD aged 10–16 years, no significant effect of remote microphone or CROS use on speech performance was found when sound was presented from the front or from monaural direct loudspeakers. However, when speech was presented via monaural indirect loudspeakers, CROS use significantly enhanced sentence recognition and comprehension. Questionnaire results revealed perceived benefits of CROS in scenarios involving speech from the front or side, but not in situations requiring sound localization. These findings support the conclusion that CROS systems may hold potential for improving auditory outcomes in classroom settings, particularly when only a single microphone is available.

### 4.3. Are CI-Implanted Patients True Binaural Listeners?

A recent study from the University of North Carolina at Chapel Hill examined spatial release from masking in cochlear-implant users. These results indicated that CI use was beneficial in diffuse-noise conditions, did not introduce detrimental interference, and may promote true binaural hearing [65]. In a favorable listening condition where speech was presented to the front and noise was directed to the deaf or implanted ear, untreated SSD patients performed as well as or better with the CI on than with the CI off. This finding suggests that CI use does not interfere with speech understanding of the normal-hearing ear and may provide an advantage derived from binaural cues such as squelch [66].

Speech perception in quiet is generally not problematic for individuals with SSD; however, hearing in background noise represents a more clinically relevant endpoint. The benefits of binaural hearing are the primary motivators for CI treatment in SSD. Testing this benefit in children presents logistical challenges because many younger children are unable to perform advanced audiometric assessments. Despite these challenges, no evidence of interference between the CI and the normal-hearing ear was found when testing sentence comprehension in noise in the bimodal condition. Among regular CI users, speech scores were greater than or equal to 70%, whereas patients with infrequent device use scored below 40% [67].

While using a CI instead of a CROS hearing aid may compromise music perception because of the artificial perception introduced by electrical stimulation, this issue can be mitigated in specialized listening environments by turning the CI off. Further studies should explore signal-preprocessing strategies to improve music perception for SSD patients using CIs.

### 4.4. Auditory Evoked Potential Studies and Binaural Processing in CI Users

CAEP studies by Yaar-Soffer et al. revealed robust P1 responses in both the normal-hearing and bilateral conditions, but delayed latencies and reduced amplitudes in the CI-only condition [67]. These findings suggest effective audibility but persistent asynchrony between acoustic and electrical inputs, which prevents the emergence of genuine binaural interaction components. This asynchrony has been identified as a cortical biomarker of auditory processing [67].

Brown et al. emphasized that true binaural hearing relies on three central mechanisms: head shadow, binaural squelch, and binaural summation [55]. These mechanisms require precise temporal and spectral synchrony between the auditory pathways of both ears. Although pediatric CI users demonstrate measurable improvements in speech perception in noise, sound localization, and subjective spatial awareness, these improvements indicate only partial restoration of binaural processing. Cortical and perceptual data suggest that while CI stimulation provides bilateral auditory input, it does not fully replicate the fidelity of natural binaural hearing. Longitudinal studies indicate that interaural synchronization improves with extended CI use and earlier implantation, suggesting ongoing neuroplastic adaptation [56].

### 4.5. SSD vs. AHL

The benefits of binaural and bimodal hearing are well established, and outcomes are particularly favorable when hearing-preservation techniques are employed. Children with AHL should be evaluated early to determine whether hearing aids provide sufficient auditory input for language development. When amplification fails or hearing continues to deteriorate, CI should be considered; improved outcomes are closely linked to early implantation, consistent with the principles of neuroplasticity [68,69]. Early assessment and timely funding approval are therefore recommended.

### 4.6. Quality of Life and Non-Audiological/Social Issues

Polonenko et al. reported consistent CI use among SSD children, including both congenital and acquired cases, with older children demonstrating longer average daily use than younger children. Importantly, CI use occurred across a wide range of everyday environments, most frequently in moderately loud settings containing speech in noise, precisely the conditions in which children with SSD may require bilateral input the most [58].

Thomas et al. described variable patterns of CI use in their cohort: sixteen subjects were full-time users, three were limited users, and one child became a non-user [70]. Among those with follow-up beyond 3 years, three of five were limited or non-users. The non-user discontinued device use after transitioning to secondary school because of lack of subjective benefit and perceived stigmatization. A stigmatization score greater than 6 on a 10-point visual analog scale was recorded in three of seventeen subjects, all older than 10 years at the time of assessment [70].

Ferguson et al. proposed a holistic, multidisciplinary model for determining CI candidacy that integrates audiological, cognitive, and socio-emotional parameters [70]. Even in healthcare systems that support bilateral implantation, unilateral CI may remain optimal when based on individualized assessment. Post-implantation outcomes from Brown et al. and Malhotra et al. demonstrated significant increases in SSQ scores, reflecting improved listening comfort, enhanced spatial awareness, and reduced cognitive effort [55,71]. Collectively, these findings indicate that CI supports not only auditory functioning but also psychosocial development and overall quality of life.

Children with AHL frequently struggle with receptive listening because of background noise and sound-localization difficulties [72]. School-aged children with unilateral hearing loss show poorer oral-language outcomes compared with their normal-hearing siblings [72]. AHL in adolescents has been negatively correlated with standardized language scores and IQ [14]. The performance gap between children with AHL and normal-hearing peers may widen with age, and when rehabilitation occurs too late, improvements in school performance may be minimal [14].

Multiple self-administered questionnaires or rating scales have been used to assess outcomes after hearing rehabilitation irrespective of device type. Arndt et al. confirmed improved subjectively perceived hearing across all three SSQ subdomains in children with post-lingual SSD treated with CI [60]. In peri-lingual SSD cases, objective outcomes did not consistently match subjective ratings, although better results were observed in children who used their CI more consistently. For the post-lingual SSD group, agreement between subjective and objective measures was robust, and all children reported daily CI use. The authors concluded that daily use is a simple but reasonably accurate indication of hearing-treatment success [60].

Doshi et al. demonstrated improvements in the Glasgow Children’s Benefit Inventory and in a visual analog scale for health status after BAHA implantation in most patients [72]. Similarly, Christensen et al. showed that outcomes assessed using HINT and the Children’s Home Inventory for Listening Difficulties improved substantially following surgery [35].

### 4.7. Language Development, Executive Function, and Cognitive Impact

The central role of hearing in supporting working memory and speech processing has been documented in multiple adult studies [73,74,75]. In the cohort from Thomas et al., weakness in academic performance or disorders of speech development constituted the primary reason for clinical referral in 11 of 21 cochlear-implanted children with SSD [68].

While some studies demonstrate improved speech and language production in bimodal children with AHL implanted in the poorer ear, relatively few investigations have examined linguistic and cognitive outcomes following SSD rehabilitation [31]. A prospective study of 25 children with different etiologies of SSD demonstrated significant deficits in speech perception, dictation, working memory, and short-term memory compared with normal-hearing controls. Rehabilitation using a transcutaneous bone-anchored implant resulted in improved performance in dictation and memory tasks in noise, and restored performance to normal-hearing levels in quiet [76].

Early CI intervention for congenital SSD appears to narrow the gap in receptive and expressive language development relative to normal-hearing peers [77]. Converging evidence shows that children with unilateral hearing loss exhibit delayed expressive and receptive language, smaller vocabulary, and reduced executive-function efficiency compared with normal-hearing children. Malhotra et al. reported significant post-implant improvements in articulation and language abilities, shown by the Goldman–Fristoe Test of Articulation and the CELF-P2 Core Language score within 12 months after surgery [70]. Notably, 93% of children demonstrated progress in at least one linguistic domain, supporting the notion that unilateral CI can partially normalize language trajectories.

Restoration of bilateral input may also enhance executive functioning by reducing auditory fatigue and freeing cognitive resources for academic and linguistic processing. However, congenital deafness and late implantation may result in persistent atypical cortical organization, potentially limiting full recovery of native-like language abilities and underscoring the importance of early auditory stimulation.

### 4.8. Study Limitations

From the review, some limitations of the studies emerge: (1) The length of post-rehabilitation follow-up between subjects and between studies was heterogeneous; long-term CI recipients may become non-users because of poor performance or stigmatization. (2) Most studies were retrospective, and patients’ age and time at testing were heterogeneous, while methods of speech-recognition testing varied between subjects and between centers. (3) Although the review included studies on both children with SSD and AHL according to the eligibility criteria [23,29,30,31,32,33,34,35,36,59,60,65,67,68,69,70,72,76,77,78,79,80,81,82,83,84,85,86,87,88,89,90,91,92,93,94,95,96,97,98,99,100,101,102,103,104,105,106,107,108,109,110,111,112,113,114,115], the current literature is predominantly focused on SSD, resulting in an underrepresentation of AHL. Consequently, conclusions regarding rehabilitation strategies for AHL should be interpreted with caution, as they are based on a much smaller body of evidence. (4) More data are needed for stronger comparisons among rehabilitation modalities, particularly with standardized pediatric outcome measures. (5) The use of a single database is a limitation of the present study; future investigations should incorporate additional databases.

## 5. Conclusions

The present systematic review of the literature shows how, in recent years, awareness of the impact that unilateral deafness can have on the child’s auditory and social development has increased thanks to the scientific evidence emerging from the literature. A growing number of studies have shown the effectiveness of rehabilitation of unilateral deafness regardless of the adopted technique; available strategies to rehabilitate binaural or bilateral hearing are reported in Table 2.

CROS systems are the easiest way, being non-surgical and reversible, to habilitate the deafened side; these systems provide good improvement for students in speech-in-noise scenarios such as classrooms but do not improve sound-source localization. BAHAs are used both for conductive hearing loss and for bone–CROS purposes. Hearing-in-noise and working-memory functions have been demonstrated to improve after BAHA rehabilitation.

CI remains the most frequently chosen modality. It has been shown to be able to restore functions of binaural hearing in acquired hearing loss or allow the ipsilateral pathway to develop in congenital deafness, provided that rehabilitation is early. Published studies suggest that early stimulation of the congenitally deaf ear by cochlear implantation may avoid permanent rerouting of auditory pathways and cross-modal plasticity. However, these results need to be confirmed by prospective studies not yet available in the literature.

## Figures and Tables

**Table 1:** Study population characteristics (N = 1622).

Etiology							
Not Reported (444)	Unknown (394)	Atresia Auris (49)	Viral (118) [CMV—VZV—Mumps]	Inner Ear Malformations/VIII CN Abnormalities (227)		Meningitis (41)	Others (316)
Type of rehabilitation							
		CI (903)	BCI (445)	CROS (54)			
Audiological evaluation tests							
	PTA (1405)	SNR dB Olsa (840)	CNC (485)	AzBio (378)	HINT (401)	BKB SiN (118)	
Quality-of-Life questionnaires							
	CHILD (114)	BAPP (47)	SSQ (364)	Others (37)			
Onset of deafness							
		Congenital (158)	Preverbal (884)	Postverbal (122)	Not reported (187)		
Age at rehabilitation							
		<4y (395)	4 < y < 12 (216)	12 > y (114)	Not reported (182)		

CMV: Citomegalovirus; VZV: Varicella-Zoster virus; CI: cochlear implant; BCI: bone conduction implant; CROS: contralateral routing of signal, CHILD: Children’s Home Inventory for Listening Difficulties; BAPP: Brief Assessment of Parental Perception; SSQ: speech spatial qualities; CNC: consonant–nucleus–consonant test. HINT: hearing-in-noise test; BKB SiN: Bamford–Kowal–Bench speech-in-noise test.

**Table 2:** Hearing rehabilitation modalities for asymmetric or complete unilateral hearing loss in children according to hearing loss.

Type of Hearing Loss	Hearing Rehabilitation Option	Ipsilateral Stimulation (Deaf Side) or Rerouting of Signal (Contralateral Stimulation)	Suggested References
AHL mild to moderate	HA: sensorineural or conductive HL	Ipsilateral: Binaural hearing	Briggs et al. 2011 [78], Fitzpatrick et al. 2019 [79]
	BCI: conductive HL		
AHL severe	HA	Ipsilateral	Russo et al. 2022 [115]
	BCI: passive (BAHA, Ponto, Alpha 2, ADHEAR), active (Bonebridge)	Bilateral stimulation: Ipsilateral (for conductive HL) and contralateral	Liu et al. 2017 [83], Hirth et al. 2021 [80]
	CI	Ipsilateral: Binaural hearing	Perkins et al. 2020 [29]
SSD	CROS devices: with HA or BCI	Rerouting: Unilateral hearing	Christensen et al. 2010 [35], Liu et al. 2017 [83]
	CI	Ipsilateral: Bilateral/binaural hearing	Benchetrit et al. 2021 [77], Park et al. 2021 [65], Brown et al. 2022 [56]

AHL: asymmetric hearing loss; SSD: single-sided deafness; HA: hearing aid; BCI: bone conduction implant; CI: cochlear implant; CROS: contralateral routing of signal.

## Data Availability

No new data were created or analyzed in this study. Data sharing is not applicable to this article.

## Supplementary Materials

The following supporting information can be downloaded at https://www.mdpi.com/article/10.3390/audiolres16050129/s1, Table S1. Newcastle–Ottawa Scale.

## Abbreviations

The following abbreviations are used in this manuscript:

SSD	Single-sided deafness
AHL	asymmetric hearing loss
CROS	contralateral routing of signal
CI	cochlear implantation
BAHA	bone-anchored hearing aid
ITD	interaural time difference
ILD	interaural level difference
UNHS	universal newborn hearing screening
CHILD	Children’s Home Inventory for Listening Difficulties
APHAB	Abbreviated Profile of Hearing Aid Benefit
THI	tinnitus handicap index
SSQ	Spatial and Qualities of Hearing Scale
PRISMA	Preferred Reporting Items for Systematic Reviews and Meta-Analyses
AMEI	active middle ear implant
HINT	Hearing-in-noise test
CAEP	Cortical Auditory Evoked Potential
