# Supplementary material for: Rehabilitation Strategies for Pediatric Single-Sided Deafness, Including Children with Asymmetric Hearing Loss: A Systematic Review
Source: Audiol Res. 2026 Sep 1;16(5):129. doi: 10.3390/audiolres16050129 (PMC13600006; doi:10.3390/audiolres16050129)
Supplement: Supplementary file 1 [file audiolres-16-00129-s001.zip › audiolres-4417640-supplementary.pdf]

Methodological Quality Assessment (Newcastle-Ottawa Scale)

Cohort studies / case series on cochlear implantation, hearing aids and bone-conduction rehabilitation in paediatric asymmetric/unilateral hearing loss. Maximum score: 9 stars (Selection 4, Comparability 2, Outcome 3).

Reference number	PMID	Title	Total Score	Selection (max 4 ★)	Comparability (max 2★)	Outcome (max 3 ★)
70	28288475	*Cochlear Implantation in Children With Congenital Single-Sided Deafness* **Thomas et al., 2017**	5/9 (Moderate)	3 ★ -- consecutive single-centre cohort (2012--2016), exposure from surgical records, baseline documented; no unexposed cohort	0 ★ -- no statistical adjustment for confounders	2★ -- structured objective tests (OlKiSa, SSQ), follow-up ≥12 months; but 7/21 (33%) lost to complete follow-up → adequacy not met
102	23222962	*Cochlear Implantation in Nontraditional Candidates: Preliminary Results in Adolescents With Asymmetric Hearing Loss* **Cadieux et al., 2013**	5/9 (Moderate)	2 ★ -- small, selected sample (5 adolescents), not consecutive; exposure/baseline well documented	0 ★ -- no control for confounders (within-subject design does not compensate for NOS)	3★ -- validated tests (CNC, HINT, BKB-SIN), follow-up ≥6 months, minimal loss (1 of 5 sessions missing)
31	24533767	*Cochlear implantation in asymmetrical hearing loss for children: Our experience* **Tzifa & Hanvey, 2013**	6/9 (Moderate)	3 ★ -- clinical programme cohort since 2008, exposure/baseline documented (audiograms)	0★ -- etiological heterogeneity not controlled for	3★ -- validated structured scales (CAPII, SIR), adequate follow-up, no losses reported among the 8 discussed
66	32065422	*Cochlear Implantation in Children with Single-Sided Deafness* **Deep et al., 2020**	5/9 (Moderate)	3 ★ -- consecutive review of CI database, clear exclusion criteria, defined audiometric baseline	0 ★ -- no statistical adjustment (age assessed descriptively only)	2★ -- standardized age-appropriate tests, mean follow-up 3.4 years; but 6/14 (43%) excluded for insufficient follow-up or aetiology → adequacy not met
60	25999052	*Cochlear Implantation in Children with Single-Sided Deafness: Does Aetiology and Duration of Deafness Matter?* **Arndt et al., 2015**	6/9 (Moderate)	3 ★ -- near-consecutive cohort (all children evaluated 2008--2014), exposure/baseline clear	1 ★ -- stratification by age of onset (congenital/perilingual/postlingual) partially controls for duration of deafness	2★ -- validated objective/subjective tests (OlSa, SSQ) with formal statistics; but only 13/20 (65%) with complete 12-month dataset
112	27256965	*Cochlear implantation in children with congenital unilateral deafness: Mid-term follow-up outcomes* **Távora-Vieira & Rajan, 2016**	5/9 (Moderate)	2 ★ -- very small, selected sample (n=3)	0 ★ -- sample too small for any control	3 ★ -- complete, validated objective battery, long follow-up (36--42 months), no losses
108	27631656	*Functional Result After Cochlear Implantation in Children and Adults With Single-sided Deafness* **Rahne & Plontke, 2016**	7/9 (High)	4 ★ -- consecutive cohort with a true comparison group (patients with bilateral hearing loss, same centre/period), exposure/baseline well documented	1 ★ -- formal statistical comparison (ANOVA) between SSD group and control group	2★ -- standardised objective measures with robust statistical analysis; but data missing for several subjects/time points in Table 2
111	27730647	*Early Outcomes After Cochlear Implantation for Adults and Children With Unilateral Hearing Loss* **Sladen et al., 2016**	7/9 (High)	3 ★ -- prospective enrolment with clear inclusion/exclusion criteria, IRB approval, complete baseline	1 ★ -- age at implantation used as a covariate in statistical models (RM-ANOVA)	3★ -- extensive validated battery (CNC, AzBio, HINT, SF-36, NCIQ, SSQ-C), pre-specified 6-month follow-up, 15/20 (75%) included with no unexplained losses
103	31478064	*Etiology and therapy indication for cochlear implantation in children with single-sided deafness* **Cushing et al., 2019**	4/9 (Low)	3 ★ -- large retrospective cohort of 118 children considered for implantation under an experimental protocol, with exposure and baseline work-up clearly documented; however, no unexposed comparison cohort	0 ★ -- descriptive retrospective analysis without control for key confounders or adjusted comparison between groups	1★ -- outcomes focus mainly on treatment decisions and diagnostic work-up rather than standardized post-implant clinical outcomes; adequacy of follow-up for outcome assessment is not established
105	31809969	*Usefulness of cochlear implantation in children with single sided deafness* **Ehrmann-Mueller et al., 2020**	5/9 (Moderate)	2 ★ -- small retrospective case series (n=7) with clearly documented exposure and baseline audiology, but selected sample and no unexposed cohort	0 ★ -- no statistical adjustment or matching for confounders; analyses are essentially within-subject descriptive comparisons	3★ -- structured audiological outcomes (speech in noise, localization, device use) assessed after implantation, with reported follow-up and no clear loss-to-follow-up among included children

Reference number	PMID	Title	Total Score	Selection (max 4 ★)	Comparability (max 2★)	Outcome (max 3 ★)
34	33079248	*Long-term results of cochlear implantation in children with congenital single-sided deafness* **Rauch et al., 2020**	5/9 (Moderate)	2★ -- retrospective single-centre case series of 11 implanted children with congenital SSD, exposure and baseline characteristics well described, but very small selected cohort and no non-exposed comparison group	0★ -- no formal control for confounding; age-group comparisons are descriptive only	3★ -- long-term follow-up with objective and subjective outcome measures (speech tests, CAP, SSQ), and non-use was explicitly reported for all subjects
76	31006171	*Longitudinal linguistic outcomes of toddlers with congenital single-sided deafness—Six with and twelve without cochlear implant and nineteen normal hearing peers* **Sangen et al., 2019**	8/9 (High)	4★ -- multicentre longitudinal cohort with an exposed CI group, an SSD non-CI comparison group, and normal-hearing peers; baseline participant characteristics and eligibility were clearly reported	2★ -- inclusion of two comparison groups with age-matched controls provides control for major prognostic differences, although residual confounding remains possible in this non-randomized design	2★ -- standardized linguistic, cognitive, and auditory outcomes were used longitudinally, but sample size is small and some missing data are acknowledged in outcome tables
29	32574479	*Treating the Individual Ear in Children: Results of Cochlear Implantation in Children With Asymmetric Sensory Hearing Loss* **Perkins et al., 2020**	5/9 (Moderate)	3★ -- retrospective chart review of 49 consecutively identified children meeting predefined unilateral implantation criteria, with exposure and baseline data documented; no unexposed cohort	0★ -- no adjustment for confounders, despite stratification by PBK/CNC testing format	2★ -- objective postoperative speech perception outcomes were assessed at 12 and 24 months, but the authors note limited postoperative bimodal data, reducing adequacy/completeness of follow-up
109	30354002	*Single-sided deafness and cochlear implantation in congenital and acquired hearing loss in children* **Ramos Macías et al., 2019**	5/9 (Moderate)	2★ -- observational descriptive study of implanted children younger than 12 years with congenital or acquired SSD, with baseline and exposure clearly described; however, the cohort is small and lacks an unexposed comparison group	0★ -- no control or adjustment for confounding between congenital and acquired cases	3★ -- postoperative outcomes included speech testing, cortical responses, lateralization testing, SSQ, and defined 12-month follow-up after processor use, with no clear attrition among reported subjects
30	30351204	*Improved speech and language development after unilateral cochlear implantation in children with a potentially useable contralateral ear* **Schaefer et al., 2018**	5/9 (Moderate)	3★ -- retrospective cohort of 57 children from a clinical programme with clear exposure and baseline assessment, but heterogeneous indications and no non-implanted comparison group	0★ -- no adjusted analysis for confounding despite marked heterogeneity in age, indication, and contralateral hearing status	2★ -- outcomes included validated measures (MSLDS, CAP, BAPP) with follow-up extending up to 13.3 years, but outcome completeness was limited because only subsets had language-age-equivalent or parental questionnaire data
33	30623849	*Cochlear implantation for single-sided deafness in children and adolescents* **Zeitler et al., 2019**	6/9 (Moderate)	3★ -- retrospective multi-institutional cohort of nine pediatric SSD recipients with clear inclusion criteria, exposure ascertainment, and baseline audiology; no separate unexposed cohort	1★ -- duration of deafness and etiology were collected and considered descriptively, but no robust adjusted analysis was feasible in this small cohort	2★ -- objective postoperative measures in quiet and noise, tinnitus, and device use were reported over a median 12.3-month follow-up, though only 6 of 9 had at least 12 months of postoperative audiometric data
65	33769866	*Spatial Release From Masking in Children With Unilateral Hearing Loss Following Cochlear Implantation* **Park et al., 2021**	7/9 (High)	3★ -- prospective clinical-trial cohort of 20 implanted children with clearly defined inclusion/exclusion criteria and complete baseline demographic/audiologic characterization; no external unexposed cohort	1★ -- within-subject CI-on versus CI-off design provides each child as their own control, but there is no independent matched cohort and limited adjustment for confounders	3★ -- standardized outcome assessment at the prespecified 12-month interval was used, and all 20 implanted subjects completed the 12-month study interval included in analysis
69	34224547	*Influence of Cochlear Implant Use on Perceived Listening Effort in Adult and Pediatric Cases of Unilateral and Asymmetric Hearing Loss* **Lopez et al., 2021**	7/9 (High)	3★ -- prospective cohort at a tertiary referral center enrolling implanted adult and pediatric participants with clearly defined exposure and repeated pre/post assessments; no separate non-exposed cohort	1★ -- within-subject longitudinal comparison over predetermined intervals provides partial control, but no adjusted parallel comparison group for pediatric unilateral cases	3★ -- validated SSQ/SSQ-C listening-effort outcomes were measured preoperatively and post-activation, with reported longitudinal follow-up across the study period
56	34542181	*Benefits of Cochlear Implantation in Childhood Unilateral Hearing Loss (CUHL Trial)* **Brown et al., 2022**	7/9 (High)	3★ -- prospective FDA-approved clinical trial of 20 children with explicit candidacy criteria and comprehensive baseline testing; no external unexposed cohort	1★ -- repeated within-subject comparisons across multiple binaural domains partially control for baseline differences, but no matched non-implanted control group	3★ -- standardized objective and subjective outcomes were measured through 12 months with clearly reported postoperative intervals and complete trial-style outcome reporting

Reference number	PMID	Title	Total Score	Selection (max 4 ★)	Comparability (max 2★)	Outcome (max 3 ★)
68	34844163	*The impact of cochlear implantation on speech and language outcomes in children with asymmetric sensorineural hearing loss* **Malhotra et al., 2022**	6/9 (Moderate)	3★ -- retrospective single-centre cohort of 26 pediatric ASHL recipients with defined inclusion/exclusion criteria and baseline speech-language assessment; no non-exposed cohort	0★ -- no formal control or adjustment for confounders in this chart-review design	3★ -- repeated postoperative speech and language outcomes were assessed at regular six-month intervals using standardized age-appropriate measures
71	35852651	*In an era of bilateral funding and changing criteria, when is unilateral cochlear implantation a better option?* **Ferguson et al., 2022**	6/9 (Moderate)	3★ -- clinical audit of all first-time pediatric implants over a defined four-year period with consecutive inclusion and clear subgrouping by candidacy profile	1★ -- comparisons across three predefined audiometric groups provide partial control for clinical heterogeneity, although no multivariable adjustment was reported	2★ -- one-year postoperative functional outcomes were examined in all groups, but the study is framed as audit data and adequacy/detail of follow-up completeness is limited
98	36728258	*Effects of Age at Implantation on Outcomes of Cochlear Implantation in Children with Short Durations of Single-Sided Deafness* **Gordon et al., 2023**	7/9 (High)	3★ -- prospective cohort of 57 children implanted for SSD with clearly reported early- and late-onset groups, defined setting, and baseline characteristics; no external unexposed cohort	1★ -- age-at-implantation group comparisons provide partial control for a key prognostic factor, though not a fully adjusted comparative design	3★ -- device use, speech perception, spatial hearing, localization, and self-report outcomes were systematically evaluated after implantation with reported longitudinal analyses
67	36864095	*A cortical biomarker of audibility and processing efficacy in children with single-sided deafness using a cochlear implant* **Yaar-Soffer et al., 2023**	6/9 (Moderate)	3★ -- cohort of 22 children with SSD using a CI, with clearly described participant characteristics and standardized electrophysiologic exposure assessment; no non-exposed comparison cohort	0★ -- no control for confounding beyond within-subject listening conditions	3★ -- objective CAEP/P1 outcomes were measured systematically across monaural and bilateral conditions for all enrolled children in a clinical setting
59	37232481	*Cochlear Implantation in Very Young Children With Single-Sided Deafness* **Patro et al., 2023**	6/9 (Moderate)	3★ -- pediatric cohort focused on very young children with SSD undergoing implantation, with defined population and baseline clinical characterization; no unexposed control group	0★ -- no robust control for confounding in the nonrandomized cohort design	3★ -- postoperative clinical outcomes were reported with structured follow-up in this focused age-defined cohort
101	36879386	*Two-Year Outcomes of Cochlear Implant Use for Children With Unilateral Hearing Loss* **Park et al., 2023**	7/9 (High)	3★ -- prospective pediatric cohort with unilateral hearing loss undergoing cochlear implantation and predefined two-year follow-up protocol; no external non-implanted control group	1★ -- within-subject longitudinal design over two years provides partial control, but no matched comparison cohort or multivariable adjustment	3★ -- two-year postoperative outcomes were systematically assessed with standardized measures and explicit follow-up interval completion
100	37466238	*Long-Term Perceived Benefit of Pediatric Cochlear Implant Users with Unilateral Hearing Loss* **Hicks et al., 2023**	6/9 (Moderate)	2★ -- retrospective/long-term follow-up cohort of pediatric unilateral hearing loss CI users with clearly identified implanted sample, but selected long-term respondent group and no non-exposed cohort	1★ -- long-term benefit analyses across the implanted cohort offer limited internal comparison, but no formal adjusted control group	3★ -- validated perceived-benefit outcomes were collected in long-term follow-up with focused outcome ascertainment among included participants
95	38047440	*Cochlear implantation in children with a long average duration of single-sided deafness* **Colasacco et al., 2023**	5/9 (Moderate)	2★ -- small pediatric cohort with prolonged average duration of SSD and defined implanted sample, but selected population and no unexposed comparator	0★ -- no formal confounder control in this descriptive cohort	3★ -- postoperative outcomes and CI use were reported for the included children over follow-up
99	38244481	*Early cochlear implantation for children with single sided deafness improves spatial hearing* **Spitzer et al., 2024**	7/9 (High)	3★ -- pediatric cohort evaluating early CI for SSD with clearly defined implanted population and structured outcome assessment; no separate non-implanted cohort	1★ -- comparison by early implantation strategy and repeated within-subject testing partially addresses prognostic variation, though no full multivariable control is evident	3★ -- spatial hearing outcomes were assessed with standardized postoperative follow-up and clearly reported benefits

Reference number	PMID	Title	Total Score	Selection (max 4 ★)	Comparability (max 2★)	Outcome (max 3 ★)
96	38314892	*Cochlear Implantation Outcomes in Pediatric Unilateral Hearing Loss: Impact of Device Use and Direct Input* **Magee et al., 2024**	7/9 (High)	3★ -- multi-institutional retrospective cohort (2018--2022) with defined inclusion/exclusion criteria and clearly documented exposure/baseline (CNC testing, datalogging, DAI use); but 11/38 (29%) excluded for missing datalogging, and no external non-implanted comparison cohort	2★ -- multivariable regression adjusted the DAI--CNC association for age at CI, duration of deafness, duration of CI use, HHP, and data log hours	2★ -- validated CNC word testing under standardized sound-booth conditions, but single time-point (best score) assessment with variable duration of CI use and follow-up completeness not clearly established across the three institutions
97	38991628	*Duration of CI use in children with prelingual single-sided deafness* **Arras et al., 2024**	6/9 (Moderate)	3★ -- pediatric cohort of children with prelingual SSD examining CI use duration, with defined implanted sample and objective use-related outcomes	1★ -- comparisons centered on duration/use patterns provide partial control for prognostic variation, but no matched non-implanted cohort	2★ -- follow-up captured duration of CI use and associated outcomes, though adequacy/completeness details are not fully established from summary information
114	40307986	*Long-Term Results in Cochlear Implantation in Single-Sided Deafness* **Macías, et al. 2025**	6/9 (Moderate)	2★ -- long-term cohort of implanted SSD patients with identifiable follow-up sample, but likely selected and without non-exposed comparison group	1★ -- longitudinal within-cohort comparisons offer limited control for baseline differences	3★ -- long-term postoperative results were the central outcome with focused follow-up reporting
86	36575571	*Effect of Hearing Device Use on Speech-in-Noise Performance in Children with Severe-to-Profound Unilateral Hearing Loss* **Griffin et al., 2022**	7/9 (High)	4★ -- controlled pediatric cohort including children with unilateral hearing loss and bilateral normal-hearing peers, with all major nonsurgical device conditions systematically assessed	1★ -- case-control structure and within-subject comparison across device conditions provide partial control, though this is not a fully adjusted multivariable analysis	2★ -- standardized BKB/R-SPACE speech-in-noise outcomes were measured rigorously, but this is a laboratory performance study rather than longitudinal clinical follow-up
89	35499947	*Transcutaneous Bone Conduction Implants in Patients With Single-Sided Deafness: Objective and Subjective Evaluation* **AlFarraj et al., 2022**	5/9 (Moderate)	2★ -- retrospective study of 11 SSD patients implanted at a tertiary center with clear pre/post exposure ascertainment, but small selected sample and no non-exposed cohort	0★ -- no control for confounders beyond simple pre/post comparison	3★ -- objective audiologic and validated subjective outcomes were compared preoperatively and postoperatively for all included patients
90	38608635	*Clinical experience of an adhesive bone conduction hearing system in children with congenital single-sided deafness* **Liu et al., 2024**	6/9 (Moderate)	3★ -- pediatric cohort of 13 school-aged children with congenital SSD plus a normal-hearing comparison group, with clearly described testing conditions and device exposure	1★ -- inclusion of an NH control group and aided versus unaided comparisons provide partial control, though confounding is not formally adjusted	2★ -- standardized speech-in-noise and localization outcomes were measured, but the report appears cross-sectional/short-term rather than a true longitudinal follow-up study
92	39970808	*Surgical and audiometric outcomes of active osseointegrated bone-conduction hearing device (Cochlear™ Osia® 2 system) placement from a tertiary paediatric centre* **Mozaffari et al., 2025**	6/9 (Moderate)	3★ -- retrospective paediatric centre review of 49 patients implanted over two years with well-documented surgical and audiometric baseline data	0★ -- no formal control group or confounder adjustment, despite mixed indications including SSD	3★ -- postoperative audiometric, speech-in-noise, complication, and satisfaction outcomes were reported with defined follow-up averaging 8.7 months
88	39972997	*Active Osseointegrated Steady-State Implant System: Surgical and Clinical Performance* **Ordoñez-Ordoñez et al., 2025**	5/9 (Moderate)	2★ -- large multicenter prospective repeated-measures study, but heterogeneous mixed-age and mixed-indication sample with SSD only as a subgroup and no dedicated pediatric SSD comparison cohort	0★ -- no confounder control specific to SSD or pediatric subgroup analyses	3★ -- surgical, gain, and subjective benefit outcomes were prospectively collected between 3 and 6 months after activation
91	36924647	*Hearing outcomes in children with single-sided deafness: Bone conduction devices vs remote microphone technology* **Chandrasekar et al., 2023**	7/9 (High)	3★ -- pediatric comparative cohort focused on children with SSD using different nonsurgical rehabilitation options, with clearly defined hearing-device exposure groups	2★ -- direct comparison of bone-conduction devices versus remote microphone technology provides meaningful control for intervention type in this nonrandomized design	2★ -- hearing outcomes were reported with structured testing, though long-term adequacy of follow-up is not fully established from available text extraction

Reference number	PMID	Title	Total Score	Selection (max 4 ★)	Comparability (max 2★)	Outcome (max 3 ★)
23	25853613	*Long-term Compliance and Satisfaction With the Baha Softband and SoundArc in Patients With Unilateral Sensorineural Deafness* **Nelissen et al, 2015**	5/9 (Moderate)	2★ -- observational cohort on long-term compliance and satisfaction in unilateral sensorineural deafness, but selected users and limited baseline comparators	0★ -- no meaningful control for confounders	3★ -- long-term compliance and satisfaction outcomes were the primary endpoints and were specifically ascertained over follow-up
35	20157065	*Update on Bone-Anchored Hearing Aids in Pediatric Patients With Profound Unilateral Sensorineural Hearing Loss* **Christensen et al., 2010**	4/9 (Low)	2★ -- small pediatric observational update of BAHA use in profound unilateral SNHL, with limited sample and no unexposed comparison group	0★ -- no control for confounding	2★ -- postoperative/postaiding outcomes and clinical impressions were reported, but follow-up structure and completeness appear limited
106	33784936	*A retrospective evaluation of minimally invasive ponto surgery (MIPS) in two pediatric centers* **French,et al., 2021**	5/9 (Moderate)	3★ -- retrospective two-centre pediatric surgical cohort with clearly defined exposure to MIPS technique and perioperative outcomes	0★ -- no comparator adjustment beyond descriptive analysis	2★ -- surgical follow-up outcomes were reported, though this is primarily a technique/safety study rather than a comprehensive functional hearing-outcomes cohort
75	30439708	*Working Memory Function in Children with Single Side Deafness Using a Bone-Anchored Hearing Implant. A Case-Control Study* **Di Stadio et al., 2019**	7/9 (High)	3★ -- pediatric case-control study including children with SSD using BAHI and comparison groups, with exposure and baseline clearly defined	2★ -- explicit case-control structure offers comparative control across groups for cognitive outcome analysis	2★ -- working-memory outcomes were systematically measured, but longitudinal clinical follow-up is limited
85	23187927	*Quality-of-Life Outcomes After Bone-Anchored Hearing Device Surgery in Children With Single-Sided Sensorineural Deafness* **Doshi et al., 2013**	5/9 (Moderate)	2★ -- small pediatric postoperative cohort with clearly identified children undergoing BAHD surgery for SSD, but selected sample and no unexposed cohort	0★ -- no control for confounding	3★ -- quality-of-life outcomes were specifically measured after surgery using structured postoperative assessment
113	40407663	*Cochlear and Bone Conduction Implants in Asymmetric Hearing Loss and Single-Sided Deafness: Effects on Localization, Speech in Noise, and Quality of Life* **Astefanei et al., 2025**	5/9 (Moderate)	2★ -- longitudinal observational study of 37 adult and pediatric CI/BCI recipients with defined outcomes, but mixed ages, mixed indications, and no dedicated unexposed cohort	0★ -- no adjusted comparison controlling for implant type or age-specific confounders	3★ -- localization, speech-in-noise, tinnitus, and SSQ12 outcomes were longitudinally assessed after implantation
93	34415079	*Hearing Aid Use in Children With Unilateral Hearing Loss: A Randomized Crossover Clinical Trial* **Benchetrit et al., 2022**	8/9 (High)	4★ -- multicentre randomized crossover trial of 37 eligible children aged 6-12 years with explicit audiometric inclusion criteria, standardized hearing-aid fitting, and well-documented baseline characteristics; each child served as their own control.	2★ -- randomized intervention order and within-subject crossover comparison controlled major baseline differences; study arms were similar at baseline and formal between-/within-arm analyses were performed.	2★ -- validated HEAR-QL, CHILD, and LIFE-R measures were collected at prespecified 6-week intervals through 26 weeks; however, analysis was per protocol, only 34/37 entered analysis, and overall survey completion was 79%, so adequacy was not fully met.
107	32574079	*Contralateral Routing of Signal Systems Can Improve Speech Recognition and Comprehension in Dynamic Classrooms* **Picou et al., 2020**	6/9 (Moderate)	3★ -- clearly characterized clinic-based sample of 20 children aged 10-16 years with limited usable hearing unilaterally, documented audiometric baseline, and standardized exposure to unaided, CROS, and remote-microphone conditions; no external unexposed cohort.	1★ -- repeated within-subject comparisons across randomized device-condition order and mixed-effects models partially controlled individual differences, but recruitment was selected and no matched parallel cohort or multivariable confounder adjustment was used.	2★ -- standardized sentence-recognition and comprehension tasks were completed by all 20 participants, with objective laboratory assessment; nevertheless, testing was cross-sectional in a single session and the subjective questionnaire applied only to six established CROS users.

Reference number	PMID	Title	Total Score	Selection (max 4 ★)	Comparability (max 2★)	Outcome (max 3 ★)
32	32502913	*Therapeutic challenges and clinical characteristics of single-sided deafness in children* **Zhan et al., 2020**	5/9 (Moderate)	3★ -- retrospective electronic-record case series covering 2008-2018 with defined pediatric SSD criteria, a relatively large congenital cohort (n=88), and documented diagnostic, imaging, treatment, and baseline characteristics; no unexposed comparison cohort.	0★ -- primarily descriptive analyses without matching or adjusted control for key confounders across observation, BCHA, CROS, conventional hearing-aid, and cochlear-implant groups.	2★ -- treatment choice, device use, and clinical follow-up were systematically abstracted, with recommended monitoring intervals; however, 39.8% lacked follow-up at at least 1 year and device-use data were frequently missing, so adequacy was not met.
110	28132094	*Unilateral hearing loss in children: a retrospective study and a review of the current literature* **Rohlfs et al., 2017**	7/9 (High)	3★ -- retrospective clinical cohort of children routinely monitored or treated for UHL, with clearly documented audiologic baseline and three exposure groups (current hearing aid, no hearing aid, previous hearing aid); the review component was not scored separately.	2★ -- direct comparison among current-, never-, and former-hearing-aid groups, which were reported as similar for age, observation period, sex, side, school type, age at diagnosis, age at fitting, and speech development.	2★ -- objective audiometry and structured parent/child questionnaires assessed hearing, device acceptance, and HRQoL; 74/87 families responded (85%), but retrospective assessment, incomplete audiometric improvement data, and no fixed prospective follow-up interval limit adequacy.
94	34661482	Functional Consequences of Poor Binaural Hearing in Children **McSweeney et al., 2021**	7/9 (High)	4★ -- cohort study with three clearly defined groups (bilateral CI, unilateral hearing loss, normal-hearing peers), recruitment setting and eligibility reported, baseline demographics described, and exposure/hearing status ascertained with standardized audiologic criteria.	2★ -- includes meaningful comparison groups and age-adjusted regression/mixed-model analyses across groups, providing control for major prognostic differences, although residual confounding remains possible in this non-randomized design.	1★ -- outcomes were measured with multiple standardized validated tests (PBK, SSQ, CELF, WIAT-III, BOT-2, memory tasks), but the design is essentially cross-sectional without a true longitudinal follow-up period for clinical outcome adequacy.
